# Evaluation of four final irrigation protocols for cleaning root canal walls

**DOI:** 10.1038/s41368-020-00091-4

**Published:** 2020-10-19

**Authors:** Qiang Li, Qian Zhang, Xiaoying Zou, Lin Yue

**Affiliations:** 1 Department of Cariology, Endodontology and Operative Dentistry, School and Hospital of Stomatology, Peking University, Beijing, PR China; 2 Central Laboratory, Peking University School and Hospital of Stomatology, Beijing, 100081 PR China

**Keywords:** Pulpitis, Root canal treatment

## Abstract

The aim of this study was to compare the efficiency of four final irrigation protocols in smear layer removal and bacterial inhibition in root canal systems. Thirty roots inoculated with *Enterococcus faecalis* were prepared with ProTaper Universal files. The teeth were disinfected by conventional needle irrigation, sonic agitation using the EndoActivator device, passive ultrasonic irrigation, or an M3 Max file. Teeth with no root canal preparation served as blank controls for the establishment of the infection baseline. Teeth with preparation but no final irrigation served as a post-instrumentation baseline. After the final irrigation, the teeth were sectioned in half. One half of each tooth was examined by scanning electron microscopy (SEM) to assess smear layer removal using a five-point scale. The other half was examined by confocal laser scanning microscopy (CLSM) using the LIVE/DEAD BackLight bacterial viability kit to evaluate the depth of bacterial survival in dentinal tubules. SEM analysis revealed no significant difference in smear layer removal throughout the whole canal among the EA, PUI, and M3 Max groups (*P* > 0.05). CLSM revealed that PUI achieved the greatest bacterial inhibition depth in the coronal ((174.27 ± 31.63) μm), middle ((160.94 ± 37.77) μm), and apical ((119.53 ± 28.49) μm) thirds of the canal (all *P* < 0.05 vs. other groups). According to this comprehensive SEM and CLSM evaluation, PUI appears to have the best infection control ability in root canal systems.

## Introduction

The main goal of endodontic treatment is to maintain or promote periapical tissue healing.^1^ In infectious root canals, chemomechanical cleaning and shaping of the root canal system to eliminate or reduce bacterial populations are key for positive endodontic outcomes.^1,2^ However, the creation of completely sterile conditions is challenging. Even when performed carefully, mechanical preparation cannot reach large areas (>35%) of the canal walls, particularly in the apical third of the root.^3,4^ Therefore, chemical irrigation is of great importance for root canal disinfection. Sodium hypochlorite (NaOCl) is the most popular and widely used chemical irrigant due to its efficacy against pathogenic organisms and pulp digestion.^5,6^ The use of ethylenediaminetetraacetic acid (EDTA) as an irrigant is often recommended because this acid can chelate and remove the mineralized portion of the smear layer.^7^ Irrigation techniques based on different agitation protocols have been developed to improve the efficacy of irrigants. The aim of such treatment is to remove the smear layer created by mechanical instrumentation on the canal wall surface, thereby promoting NaOCl penetration to kill bacteria that deeply colonize the dentinal tubules.^8^

Passive ultrasonic irrigation (PUI) is more efficient than conventional needle irrigation (CNI) in the removal of debris^9^ and the smear layer^10^ because of acoustic streaming and cavitation.^11^ The sonically driven EndoActivator (EA) canal irrigation system (Dentsply, York, PA, USA) uses disposable flexible polymer tips of different sizes. The activator tips can be operated at 2 000–10 000 cycles per min without damaging the root dentin. The nickel–titanium (NiTi)-based M3 Max irrigation file (United Dental, Shanghai, China) was recently introduced in China. According to the manufacturer, the M3 Max instrument is similar to the XP-Endo Finisher (FKG, Switzerland) in terms of its application and properties. This ISO 25/.01 file has a unique spoon shape, with a length of 10 mm from the tip and a depth of 1.5 mm. The manufacturer recommends its operation with vertical motions at 600 r·min^−^^1^ with 1 N·cm torque to “scrape” the root canal walls, thereby disturbing the smear layer or biofilm.

Generally, thorough disinfection of root canal systems should include not only smear layer removal but also inhibition of bacterial colonization deep in dentinal tubules.^12–14^ Many studies have investigated the ability of final irrigation to remove the smear layer,^15–19^ but few have evaluated its ability to inhibit bacterial growth in dentinal tubules.^20^ Moreover, previous studies have examined smear layer removal or bacterial inhibition separately; thus, the relationship between smear layer removal and bacterial inhibition remains unclear. A comprehensive three-dimensional assessment of the cleaning abilities of different final irrigation protocols is lacking. Therefore, the aim of this study was to evaluate the cleaning effect of four different irrigation protocols (CNI, EA, PUI, and M3 Max). The combined application of scanning electron microscopy (SEM) and confocal laser scanning microscopy (CLSM) in the same root canal systems can help to reveal the relationship between smear layer removal and bacterial inhibition achieved with different irrigation protocols and the underlying mechanisms.

## Results

### Efficiency of smear layer removal from root canal surfaces

**Fig. 1:**
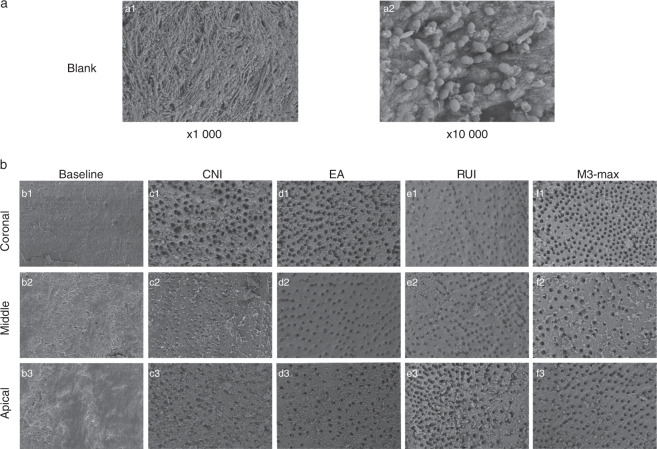
Representative scanning electron microscopy images showing smear layers on canal surfaces of different groups. **a** Upper panel: images from the blank control group at ×1 000 and ×10 000 magnification confirming *E*. *faecalis* incubation (**a1**, **a2**). **b** Lower panel: images of the coronal, middle, and apical canal thirds from the post-instrumentation baseline (**b1–b3**), conventional needle irrigation (CNI; **c1–c3**), EndoActivator (EA; **d1–d3**), passive ultrasonic irrigation (PUI; **e1–e3**), and M3 Max (**f1–f3**) groups. ×1 000 magnification

Representative images of smear layers in the coronal, middle, and apical regions from the different groups are shown in Fig. 1. Images from the blank control group taken at magnifications of ×1 000 and ×10 000 confirmed *Enterococcus faecalis* incubation. Representative images depicting middle canal regions are shown in Fig. 1Ia1, a2. *E*. *faecalis* can be visualized as clusters or short chains and strings adhering to the root canal walls under ×10 000 magnification. Smear layer scores are shown in Table 1. Overall, they were significantly lower in the experimental groups than in the baseline group (*P* < 0.05). The mean score of the smear layer for the CNI group (3.71 ± 0.46) was significantly higher than those for the other three experimental groups (3.25 ± 0.47 for the EA group, 3.00 ± 0.77 for the PUI group, and 2.96 ± 0.71 for the M3 Max group). No significant differences were observed among the EA, PUI, and M3 Max groups. Smear layer scores were higher in the apical region than in the middle and coronal regions in all experimental groups. The mean score for the coronal region was lowest in the M3 Max group (2.40 ± 0.51); it was 3.02 ± 0.41 in the EA group, 2.67 ± 0.62 in the PUI group, and 3.47 ± 0.52 in the CNI group (*P* < 0.05). Pairwise comparison revealed no significant difference between the EA and PUI groups. The other pairwise comparisons indicated significant differences. The mean scores for the middle region were 3.33 ± 0.49 in the EA group, 2.80 ± 0.78 in the PUI group, and 2.87 ± 0.52 in the M3 Max group, which were significantly lower than that in the CNI group (3.67 ± 0.49; *P* < 0.05). Pairwise comparisons showed no significant differences between the EA, PUI, and M3 Max groups. The mean score for the apical region was lowest in the EA group (3.40 ± 0.51); it was 4 in the CNI group, 3.53 ± 0.64 in the PUI group, and 3.60 ± 0.51 in the M3 Max group (*P* < 0.05). Pairwise comparisons revealed a significant difference between only the EA and CNI groups (*P* < 0.05).

**Table 1:** Mean value ± standard deviation of smear layer scores of different groups

Items	Baseline	CNI	EA	PUI	M3 Max
Overall	5^a^	3.71 ± 0.46^b^	3.25 ± 0.47^c^	3.00 ± 0.77^c^	2.96 ± 0.71^c^
Coronal	5^d^	3.47 ± 0.52^e^	3.02 ± 0.41^f^	2.67 ± 0.62^f^	2.40 ± 0.51^g^
Middle	5^h^	3.67 ± 0.49^i^	3.33 ± 0.49^j^	2.80 ± 0.78^j^	2.87 ± 0.52^j^
Apical	5^k^	4^l^	3.40 ± 0.51^m^	3.53 ± 0.64^l^	3.60 ± 0.51^l^

Different lowercase letters indicate significant differences between groups (*P* < 0.05)

Mean value ± standard deviation of smear layer scores after conventional needle irrigation (CNI), sonic agitation with the EndoActivator device (EA), passive ultrasonic irrigation (PUI), and agitation with the M3 Max file (M3 Max)

### Efficiency of bacterial inhibition in dentinal tubules

**Fig. 2:**
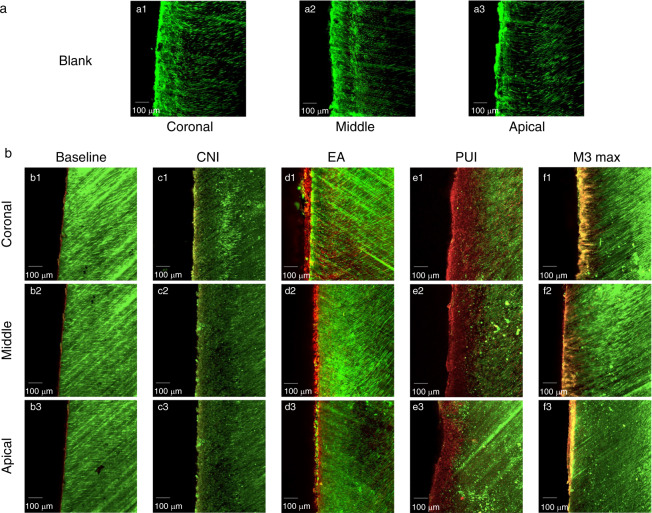
Representative confocal laser scanning microscopy images showing bacterial inhibition in dentinal tubules of different groups. **a** Upper panel: images of the blank control group with only green fluorescence (live bacteria) to confirm the *E*. *faecalis* incubation (**a1–a3**). **b** Lower panel: Confocal laser scanning microscopy images of live (green) and dead (red) bacteria in dentinal tubules of the coronal, middle, and apical canal thirds from the post-instrumentation baseline (**b1–b3**), conventional needle irrigation (CNI; **c1–c3**), EndoActivator (EA; **d1–d3**), passive ultrasonic irrigation (PUI; **e1–e3**), and M3 Max (**f1–f3**) groups

**Fig. 3:**
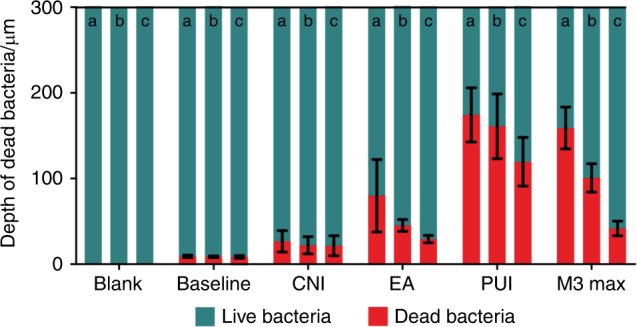
Quantitative analysis of dead bacteria in dentinal tubules detected by confocal laser scanning microscopy. Graphs show the depth of dead bacteria within 300 µm in the dentinal tubules after the use of different irrigation techniques. a, coronal third; b, middle third; c, apical third

Representative images of bacteria in dentinal tubules are presented in Fig. 2. The depth of green fluorescence exceeded 300 μm in all root canal walls. The blank control group showed only green fluorescence, with no red fluorescence in the dentinal tubules (Fig. 2a1–a3). In contrast, the depths of red fluorescence in the tubules differed between the baseline group and the four experimental groups (Fig. 2b1–b3, c1–c3, d1–d3, e1–e3, f1–f3). The depths of the measured red fluorescence in different groups are shown in Table 2 and Fig. 3. The data were not distributed normally and were analyzed using nonparametric statistical tests. The Kruskal–Wallis test revealed significant differences among groups, with the PUI group showing the greatest depth of bacterial inhibition in dentinal tubules. Pairwise comparisons also revealed significant differences.

**Table 2:** Mean value ± standard deviation (µm) of red fluorescence of different groups

Items	Blank	Baseline	CNI	EA	PUI	M3 Max
Overall	0^a^	8.71 ± 1.39^b^	22.16 ± 11.33^c^	51.46 ± 32.23^d^	151.58 ± 39.51^e^	100.45 ± 51.68^f^
Coronal	0^g^	9.05 ± 1.53^h^	26.64 ± 12.53^i^	79.94 ± 42.37^j^	174.27 ± 31.63^k^	158.91 ± 24.37^l^
Middle	0^m^	8.65 ± 1.12^n^	19.12 ± 10.01^o^	45.20 ± 7.02^p^	160.94 ± 37.77^q^	100.79 ± 16.65^r^
Apical	0^s^	8.43 ± 1.57^t^	21.51 ± 11.68^t, u^	29.24 ± 4.39^u^	119.53 ± 28.49^v^	41.66 ± 8.66^w^

Different lowercase letters indicate significant differences between groups (*P* < 0.05).

Mean value ± standard deviation of red fluorescence after conventional needle irrigation (CNI), sonic agitation with the EndoActivator device (EA), passive ultrasonic irrigation (PUI), and agitation with the M3 Max file (M3 Max).

The depths of red fluorescence in the apical region were 0 μm (blank control), (8.43 ± 1.57) μm (post-instrumentation baseline), (21.51 ± 11.68) μm (CNI), (29.24 ± 4.39) μm (EA), (119.53 ± 28.49) μm (PUI), and (41.66 ± 8.66) μm (M3 Max). Pairwise comparison revealed no significant difference between the CNI group and the baseline or EA group but significant differences for all other comparisons (*P* < 0.05). Similarly, the depth of bacterial inhibition in the middle third of the canal was greatest in the PUI group ((160.94 ± 37.77) μm), followed by the M3 Max ((100.79 ± 16.65) μm), EA ((45.20 ± 7.02) μm), and CNI ((19.12 ± 10.01) μm) groups. Pairwise comparisons revealed significant differences between all groups in the middle third of the canal (*P* < 0.05). The PUI group also showed the greatest bacterial inhibition in the coronal third ((174.27 ± 31.63) μm) of the canal. Pairwise comparisons revealed significant differences between all comparisons in the coronal third (*P* < 0.05).

## Discussion

This study compared the efficacy of four irrigation protocols in terms of smear layer removal and lateral penetration into the dentinal tubules to kill bacteria. Combined SEM and CLSM analysis of the same root canal systems allowed us to demonstrate the relationship between smear layer removal from the canal wall surfaces and the bactericidal effect deep in the dentinal tubules.

Ideally, chemomechanical preparation should thoroughly clean and disinfect the root canal system. Previous studies have shown that preparation of the apical third of the canal and the depth of irrigant penetration into the root canal system play key roles in the realization of this goal.^21,22^

No differences in debris removal have been found between PUI and the use of XP-Endo Finisher.^23^ In addition, compared to PUI and XP-Endo Finisher use, EA use does not result in significantly different debris and smear layer removal in single-rooted teeth.^24,25^ We did not find a significant difference in smear layer removal among the PUI, M3 Max, and EA protocols, consistent with previous studies. The M3 Max is an ISO 25/.01 instrument with a recommended use speed of 600 r·min^−^^1^, whereas the XP-Endo Finisher is an ISO 25/.00 instrument with a recommended minimum use speed of 800 r·min^−1^. Whether these differences in taper and optimal speed result in different smear layer removal abilities are unknown. Further research is needed to evaluate the differences between the M3 Max and XP-Endo Finisher systems.

We found that the M3 Max protocol achieved the best smear layer removal in the coronal region, which was attributable to the ductility and flexibility of the M3 Max files. The depth of the spoon-shaped part of the M3 Max file exceeds 1.5 mm, enabling full contact with the root canal walls and scraping of the smear layer in the coronal region, which then was flushed out with the irrigants. By contrast, compared to the EA treatment, the PUI treatment did not show improved performance in the coronal area. Although ultrasonic irrigation has been claimed to be effective within 3 mm of the file,^26^ the acoustic cavitation decreases markedly with increased distance from the file.^27^ In the apical region, the EA protocol achieved better results than the PUI and M3 Max protocols, which may be related to the frequency and amplitude of sonic irrigation. The maximum oscillation amplitude occurs at the activator tip, which is located in the apical third of the canal during treatment. Compared to the ultrasonic irrigator, the EA device has a lower frequency (10 kHz) and higher amplitude, resulting in greater irrigant energization.^28^ In the narrow apical region, the high-energy irrigant easily makes contact with the root canal walls, improving its cleaning ability.^20,29^ Moreover, root canal cleaning ability decreased from the coronal to the apical area in the EA, PUI, and M3 Max groups. Further exploration is needed to improve the efficiency of irrigation in the apical area. All activation groups (EA, PUI, and M3 Max) showed better smear layer removal efficacy than did the CNI group, which was not treated with activation. This result is consistent with those of previous studies^17,20^ and confirms the necessity of using activation with irrigation. However, no significant difference was found among the EA, PUI, and M3 Max groups, which may be related to the limited sample size of this study.

As this study focused on the depth of bacterial inhibition, CLSM was used to intuitively identify the locations of the living and dead bacteria in the tubules. According to the CLSM results, the depth of green fluorescence exceeded 300 μm in all root canal walls, confirming the successful establishment of the *E*. *faecalis* infection model and the comparability of all roots. CLSM analysis also showed the presence of dead bacteria in the dentinal tubules of all groups except the blank control group, verifying the bacterial inhibition ability of NaOCl.^8^ Differences in the depths of bacterial inhibition among groups suggested that the efficiency of NaOCl penetration into dentinal tubules differed with group. Zou et al.^8^ showed that NaOCl can penetrate dentinal tubules to 77–300 μm. Thus, we compared the depths of bacterial inhibition at 300 μm. The depth was greatest in the PUI group, which suggests that acoustic streaming and cavitation contribute to increased penetration of irrigants into dentinal tubules.^27^ The depth of bacterial inhibition was lower in the EA group than in the PUI group. Previous research has also demonstrated that PUI promotes significantly more irrigant penetration than EA treatment.^30^ The poor acoustic cavitation effect produced by sonic instruments may affect the penetration of irrigants^31^. In a previous study, an XP-Endo Finisher protocol showed better bacterial inhibition ability than EA treatment at 50-µm depth in dentinal tubules but markedly decreased effects at 100- and 150-µm depths.^20^ In our study, the M3 Max, a NiTi file agitation system similar to the XP-Endo Finisher, also showed better bacterial inhibition ability than the EA device. The mechanical scraping of the M3 Max file during operation, together with its agitation of the irrigant, may facilitate removal of the smear layer from the canal walls, further promoting the penetration of NaOCl into the dentinal tubules to kill bacteria. Notably, PUI and M3 Max achieved similar smear layer removal from root canal surfaces, but PUI appears to have the best disinfecting effect deep in dentinal tubules. This difference may stem from the greater ultrasonic intensity of PUI, which may result in a greater amplitude of oscillation and enhanced cleaning efficacy.^32^ It may allow deeper NaOCl penetration in dentinal tubules after removal of the smear layer from the surfaces of the canal walls. However, the M3 Max may have less effect on the activation of the irrigants in the canal space, resulting in reduced irrigant penetration efficiency. Still, the mechanical scraping effect resulting from the file’s unique spoon shape may effectively improve the disinfection effect on the root canal surface.

## Conclusions

Based on the morphological observations supported by the sample size provided in this study, we can conclude that PUI appears to have the best disinfection ability in root canal systems. Although M3 Max achieved better smear layer removal in the coronal region because of the scraping effect, its bacterial inhibition ability deep in the dentinal tubules was unsatisfactory. Further studies are required to determine the efficiency of these protocols in canals with different anatomies.

## Materials and methods

### Tooth selection and preparation

Thirty freshly extracted intact premolars with straight root canals and no apical resorption were collected from the clinic of the Department of Oral and Maxillofacial Surgery and placed in physiological saline. Teeth with a history of restoration or endodontic treatment were excluded. This study was approved by the Institutional Review Board of Peking University’s School of Stomatology (PKUSSIRB-201629073). The sample size was determined using a completely randomized design^33^ performed with PASS for Windows software (ver. 15.0; NCSS Inc., Kaysville, UT, USA). With a confidence coefficient of 0.95 (*α* = 0.05) and a power of 0.85 (*β* = 0.15), the minimum sample size for SEM analysis was calculated to be 5 in each group, while the minimum sample size for CLSM analysis was 3 in each group. Thus, the total sample size was determined to be 30 (5 in each group) for both SEM analysis and CLSM analysis. The teeth were decoronated using a water-cooled high-speed bur (Mani, Tochigi, Japan). According to a previously described protocol,^34^ a #10 K-file (Mani, Tochigi, Japan) was inserted into the canal until the file tip was visualized at the apical foramen. Then, the roots were shortened under guidance of the K-file stopper, which was set at 12 mm. The working length (WL) of the root canal was set to 11 mm (1 mm short of the apical foramen). *E*. *faecalis* infection was established prior to root canal preparation to mimic the clinical scenario of root canal infection, following a model proposed in a previous study.^35^ The pulp tissue was removed using a barbed broach (Mani, Tochigi, Japan). Then, the teeth were autoclaved at 121 °C and 15 MPa for 20 min. A standard suspension of *E. faecalis* (29212; American Type Culture Collection, Rockville, MD, USA) was prepared from a 24-h bacterial culture in brain heart infusion (BHI; Oxoid, Basingstoke, England) at 37 °C, with spectrophotometric adjustment to ensure that the bacterial count was 1 × 10^8^ cells per mL. The root canals were filled with *E*. *faecalis* suspension to the orifice level using a 30-gauge side-vented needle (Dentsply Tulsa Dental, Tulsa, OK, USA). The roots were incubated in 10-mL BHI broth at 37 °C for 3 weeks to allow bacterial colonization on the canal walls and in the dentinal tubules. Fresh culture medium was supplied every 3 days. After incubation, the apical foramens were sealed with flowable composite (Ivoclar Vivadent, Schaan, Liechtenstein). Roots in the blank control group were not instrumented after incubation. The other 25 roots were prepared using ProTaper Universal instruments (Dentsply Maillefer, Switzerland), starting with Sx and continuing through the sequence S1, S2, F1, F2, and F3. The WL of each root was 11 mm, and the final working width was 30#. During root canal preparation, the canals were irrigated with 2 mL 5.25% NaOCl solution through a 30-gauge side-vented needle (Dentsply Tulsa Dental, Tulsa, OK, USA) between each file change.

### Final irrigation protocols

After preparation, the teeth were randomly divided into six groups (*n* = 5/group). In group 1 (blank control), the root canals were not instrumented after incubation to allow establishment of the baseline for infection before root canal preparation. In group 2 (post-instrumentation baseline), the root canals were prepared as described above, but no final irrigation was performed. In the four experimental groups (3.1–3.4), final irrigation was performed after root canal preparation using CNI (group 3.1), EA (group 3.2), PUI (group 3.3), or M3 Max (group 3.4). Each canal was irrigated with 2 mL 5.25% NaOCl for 1 min (2 mL per min), followed by 2 mL 17% EDTA (2 mL per min), and 2 mL sterilized water (2 mL per min) to remove the residual irrigant. The irrigants were agitated according to the irrigation protocols.

### Group 3.1: CNI

CNI was performed with a disposable syringe and a 30-gauge side-vented needle (Dentsply Tulsa Dental, Tulsa, OK, USA). Each canal was flushed with a continuous flow of 2 mL NaOCl for 1 min within 1 mm of the WL using a vertical motion. Then, 2 mL 17% EDTA was flushed into the canal for 1 min within 1 mm of the WL. Finally, 2-mL sterilized water was flushed into the canal using the same method. A rubber stopper was used for WL control.

### Group 3.2: EA

The canal was passively filled with irrigant. An irrigation needle was placed at the orifice level. Under constant irrigation, a red (25/04) EA tip was placed in the canal 1 mm short of the WL and operated at a speed of 10 000 cycles per minute.

### Group 3.3: PUI

Similar to the EA procedure, the canal was passively filled with irrigant, which was activated using a PUI device (Satelec Acteon Group, Merignac, France) at the power setting of 6 out of 20 for 1 min. A 20# ultrasonic file (Satelec Acteon Group) was placed 1 mm short of the WL and operated with a vertical motion.

### Group 3.4: M3 Max

Using the same method as in the EA group, an M3 Max file (United Dental, Shanghai, China) was placed 1 mm short of the WL after the canal had been filled with irrigant. The file was operated for 1 min at 600 r·min^−1^ and 1N·cm torque using vertical motion. A rubber stopper was used for WL control.

Paper points were not used to remove the irrigants to avoid any additional influence of the distribution of the smear layer on the canal walls. All samples were placed in a solution of physiological saline and stored at 4 °C until sectioning.

### Tooth sectioning and preparation for evaluation

Using a diamond bur (MANI, Tochigi, Japan), two vertical grooves were made along the long axis of each tooth without damaging the root canal. A great-taper gutta-percha cone (Coltene-Whaledent, Allstetten, Switzerland) was placed into the root canal to facilitate the visualization of groove depth and to prevent debris pollution. Then, each root was split in half using a chisel. One half of the root was used for SEM analysis, and the other half was used for CLSM analysis. For SEM analysis, the specimens were fixed in 2.5% glutaraldehyde solution for 1 week, dehydrated in a graded series of ethanol solutions, critical point dried, coated with gold, and examined under a scanning electron microscope (S8010; Hitachi, Tokyo, Japan). For CLSM analysis, the specimens were stained for 15 min using the LIVE/DEAD BackLight bacterial viability kit (Molecular Probes, Inc., Eugene, OR, USA) and then examined under a confocal laser scanning microscope (LSM 710; Carl Zeiss, Oberkochen, Germany).

### SEM evaluation

Each specimen was first viewed at low magnification (×30) to provide an overview. As the WL was 11 mm, the apical, middle, and coronal thirds of the canal were defined 0–4, 4–8, and 8–11 mm, respectively, from the apical foramen. A location near the longitudinal midpoint of each third was selected and photographed at ×1 000 magnification and 5.0 kV. Then, two additional images were captured 1-mm coronal and 1-mm apical of this site. In total, nine images of each sample were captured.

Two practitioners who were blinded to group assignment and final irrigation procedures assessed the images. The practitioners were experienced in qualitative analysis of SEM images of root canals. According to the 2008 guidelines for the interpretation of kappa values, a kappa coefficient over 0.75 can be considered to represent excellent or good agreement.^36^ The kappa value in this study was 0.772. Root canal surfaces were scored using the standard described by Caron et al.,^17^ which was based on that developed by Hulsmann et al.:^37^ 1), no smear layer and dentinal tubules open; 2), small amounts of scattered smear layer and dentinal tubules open; 3), thin smear layer and dentinal tubules partially open (characteristic crescent appearance); 4), partial coverage by a thick smear layer; and 5), total coverage by a thick smear layer. In total, 270 images were assessed.

### CLSM evaluation

Samples were observed at 20× with an additional 2× zoom. The wavelength was set at 480/500 nm for SYTO 9 (fluorescent green nucleic acid stain) and at 490/635 nm for propidium iodide (fluorescent red nucleic acid stain) for the observation of live and dead bacteria, respectively. Nine images of each specimen were captured using the same protocol as for SEM evaluation. The width of red fluorescence (dead bacteria) at 300 µm was measured using Zen 2 software (Carl Zeiss, Oberkochen, Germany) and used to calculate the depth of bacterial inhibition in each third of the canal.

### Statistical analysis

SEM and CLSM data were analyzed using the Kruskal–Wallis test and the Mann–Whitney rank sum test for pairwise comparisons. The significance level for all statistical analyses was set at *α* = 0.05. The statistical analysis was performed with the SPSS for Windows software package (ver. 20.0; SPSS Inc., Chicago, IL, USA).
